# Bullying victimization among adolescents: Prevalence, associated factors and correlation with mental health outcomes

**DOI:** 10.1371/journal.pone.0299161

**Published:** 2024-03-18

**Authors:** Mariem Ghardallou, Ahlem Mtiraoui, Dorra Ennamouchi, Amel Amara, Amel Gara, Maha Dardouri, Chekib Zedini, Ali Mtiraoui

**Affiliations:** 1 Faculty of Medicine of Sousse, Department of Community Medicine, Research Laboratory LR12ES03, University of Sousse, Sousse, Tunisia; 2 Faculty of Medicine of Sousse, Department of Psychiatry, Farhat Hached Hospital, Research Laboratory LR12ES04, University of Sousse, Sousse, Tunisia; 3 Faculty of Medicine of Sousse, Department of Community Medicine, University of Sousse, Sousse, Tunisia; West University of Timisoara: Universitatea de Vest din Timisoara, ROMANIA

## Abstract

**Introduction:**

Knowledge of the risk factors of bullying victimization in adolescents is crucial for the implementation of preventive measures. This study aimed to determine the prevalence and associated factors of bullying victimization and to identify its correlation with mental health outcomes among middle school students in Tunisia.

**Methods:**

A cross-sectional study was conducted using a multi-stage cluster sampling technique to recruit a sample of 1111 students from 10 middle schools in El kef (Tunisia). The revised Olweus Bully/Victim Questionnaire was used to assess the prevalence and types of bullying victimization and the perceived efforts of others to counteract bullying. The Strengths and Difficulties Questionnaire (SDQ) was used for screening emotional and behavioral problems. Multivariate logistic regression analysis was conducted to determine associated factors of bullying victimization. Additionally, we tested whether emotional and behavioral problems were present for bullying victims.

**Results:**

The findings reported that 45.8% (95%CI = 45.5–46.0), of the total number of participants experienced school bullying victimization. Multivariate logistic regression analysis, revealed that repeating a grade (OR = 1.82, 95%CI = 1.31–2.54), having a working father (OR = 17.68; 95%CI = 2.29–136,15), and having a working mother (OR = 1.88, 95%CI = 1.39–2.53) were the factors significantly associated with bullying victimization. Nevertheless, a higher mother’s educational level (OR = 0.76, 95%CI = 0.67–0.88) was a protective factor against bullying victimization. The self-reported SDQ revealed that the total difficulties score was significantly higher among victims (17.46 ± 5.30 vs. 20.86 ± 5.06, p<0.01).

**Conclusions:**

This study showed that the prevalence of bullying in middle schools was high and it significantly led to mental health problems. National policies for bullying prevention within schools are potentially needed. Improving students’ problem-solving and soft skills is also essential.

## Introduction

Bullying is a global public health problem [1]. It is a kind of aggressive behavior occurring mainly within middle schools in early adolescence (10 to 13 years) [2]. The transition from elementary to middle school is an important stage in the development of young adolescents. During this critical period, young people are dealing with new peer groups and can use bullying as a means of social control [3].

Bullying behavior has been defined as deliberate aggressive behavior repeated over a period of time, where there is an imbalance of power between the bullying victim and the perpetrator [4].

Bully victimization (BV) refers to the process by which an adolescent is repeatedly and over time exposed to intentional negative actions by their peers, and it can include physical, verbal, relational or social aggression [5]. The International Sustainable Development Goal Thematic Indicator 4.a.2 (2018), which measures the ‘percentage of students who experienced bullying during the past 12 months’, considered a student as victim of bullying when the frequency of aggressions is at least once or twice a month or more for a student [6].

The United Nations Educational, Scientific and Cultural Organization (UNESCO) reports an overall bullying victimization (BV) rate of 32% in 11-year-old pre-adolescents [6]. There are significant differences between regions. Recently, and according to data from the Global School Student Health Survey (GSHS) of pre-adolescents and adolescents aged 12–17 in 83 low- and middle- to high-income countries in the six World Health Organization (WHO) regions, the pooled prevalence of bullying victimization on one or more days in the previous 30 days was 30.5%. The highest prevalence was reported in the Eastern Mediterranean (45.1%) and Africa (43.5%), while the lowest prevalence was found in Europe [6,7].

North Africa is the world’s second most prevalent region for bullying, with 42.7% of students reporting having been bullied at least once in the past month (from 30.6% to 70%) [6].

In Tunisia, according to the GSHS conducted in 2008, around half (46.4%) of students report having been physically assaulted and 37.7% of students have been verbally assaulted one or more times in the last 12 months [8]. However, a survey conducted in 2015 in the region of Sousse (Tunisia) and using the revised Olweus Bully/Victim Questionnaire (OBVQ), reported that 11.7% of middle-school students are victims of bullying [9].

The adoption of appropriate methods for assessing BV in schools enables the extent of the problem to be properly estimated within the educational context, so that comprehensive school-wide anti-bullying programs can be understood, planned and evaluated [10].

A scientifically valid assessment of school bullying involves systematic observations of the frequency, duration and form of bullying in various settings, and/or the administration of reliable and valid surveys and questionnaires [10]. The Olweus Bully/Victim Questionnaire is an example of a valid and widely used self-evaluation tool. It has been used in different countries and translate in different languages [11].

Bullying victimization is a behavior that may be a form of adaptation to unfavorable ecological systems. In the microsystem, it can be influenced by individual characteristics (age, gender, personality, etc.), family characteristics (socio-economic status, parent educational level, parent employment,.) and direct relationships with parents and peers. Beyond the microsystem, mesosystem factors or interactions between social relationships (home, school) can encourage bullying. These interactions can be part of the exo-system (Community) and, further afield, the macro-system (cultural and political norms) [12,13].

It is essential to identify factors that influence vulnerability to bullying in order to track down children who are at high risk of being bullied and therefore to implement appropriate prevention strategies.

Many studies have examined the adverse health and psychosocial problems associated with bullying victimization. The most commonly reported ones are mental health problems. Symptoms often begin slowly and are non-specific, and not all victims respond to bullying in the same way [14]. Being bullied may result in internalizing problems for some people (i.e., self-harm) leading to deleterious mental health conditions, such as depression, and anxiety [15,16]. Others may experience externalizing sequelae (i.e., being harmful to others), including violent behavior toward others, carrying a weapon, and becoming a perpetrator of bullying behaviors [17]. In addition to mental health outcomes, it was reported that school bullying is also associated with higher rates of risk-taking behaviors such as smoking, alcohol and cannabis use, and earlier sexual experience [6].

School-based mental health screening is also a priority issue for assessing the effect of BV on young people. The use of a brief, simple and easy-to-use management tool for adolescent populations is strongly recommended.

As research on bullying victimization in North African countries, and particularly in Tunisia is scarce, it is essential to conduct research on BV among middle school adolescents while using a reliable and valid tool and the factors associated with this problem, in order to recognize and address this issue. Furthermore, the consequences of bullying on adolescent mental health remain little studied. Therefore, examining bullying behaviors and their impact on adolescent mental health in a sample of Tunisian students would yield interesting results. These results will help policy-makers and educators to build appropriate interventions.

The main aim of this study was therefore two-fold: (a) to investigate the prevalence, types and culture of bullying victimization among a sample of middle school adolescents in Tunisia using the Olweus Bully/Victim questionnaire (b) to examine the socio-demographic and academic characteristics of those who were exposed to or experienced bullying. In addition, the correlation of bullying with mental health of victims was studied. To this end, we examined participants’ experiences of emotional and behavioral disorders (measured by the Strengths and Difficulties Questionnaire (SDQ).

The results of this study should give us a better understanding of bullying victimization in middle schools, and enable all stakeholders to develop sustainable interventions to effectively prevent or reduce bullying in order to create a safer learning environment. In addition, this study will make a significant contribution to the literature on bullying on middle schools across all cultures.

## Methods

### Study design

A cross-sectional study was conducted during the Academic year 2018/2019 among middle school students in the governorate of El Kef.

### Study setting

The governorate of El Kef is one of the twenty-four governorates of Tunisia. It is located in central-western Tunisia. It comprises 12 delegations. Only 7 delegations were selected randomly for this study. These 7 delegations comprise 26 public middle schools. All middle schools were mainly located in urban or semi-urban areas. There are no middle schools in rural areas in Tunisia. Under the Tunisian education system, middle education consists of the last three years of preparatory education, which take place at middle school or college (age 12–14). The education rate in this region was estimated to 95,2% in 2018 [18].

### Participants

The study population included students who were attending middle schools during the Academic year 2018/2019 in the governorate of El Kef.

#### Inclusion criteria

All students regularly attending public middle schools in this region and presenting on the day of the survey were included.

#### Exclusion criteria

Parental or student refusal to participate was an exclusion criterion in the current study.

### Sampling

#### Sampling size

A cluster sampling method was used to select a random sample and the sample size was calculated according to the following formula: n = Z_α/2_^2^ × (p × q) / i^2^ (n = minimum sample size, Z_α/2_ = 1.96 (α = 0.05), p = 0.12; q = 0.88, i = 0.03). At a 95% confidence interval and assuming a problem frequency of 12% [9] and a 5% margin of error, the minimal sample size required was 451 school students. A design effect of 2 was used to compensate for the error of the estimate encountered using cluster sampling (instead of simple random sampling) and 20% was added to compensate for potential non-response. The final total sample size was estimated to be 1100 students.

#### Sampling method

The sample was obtained as follows. First, the number of clusters (classes) was determined. According to the local education directorate, the mean number of students per class was 24. Thus, the number of clusters required was 46 (the sample size divided by the mean number of students per class = 1100/24). Secondly, the required number of public schools was determined. According to the local education directorate, there were 26 middle schools in the governorate and the number of classes per middle school varies from 5 to 30. A total of 46 classes were selected from 10 middle schools, with 5 classes from each one. Then, 10 middle schools were randomly selected. Finally, based on the list of classes in each middle school, 5 classes were selected among those having a course that day.

### Data collection

A brief questionnaire was used to collect the socio-demographic and academic characteristics, such as gender, age, family members, whether living with both parents or not, place of residence, student’s rank in the family, parental educational level, parental working status, school grade, grade repetition, leisure activity practice, smoking, alcohol consumption and substance use.

The Arabic version of the revised and self-reported Olweus Bully/Victim Questionnaire (BVQ) was used to assess bullying victimization [9,19]. Only items on victimization were considered for this study (18 items). These items assess the frequency and types of bullying, the frequency of reporting bullying incidents to teachers or families, and whether teachers intervene when bullying occurs in the previous couple of months. The students responded to all the items on a 5-point scale: “never”, “only once or twice”, “2 or 3 times a month”, “about once a week”, or “several times a week”. According to Solberg and Olweus, the standard cutoff point for classifying a student as a victim or non-victim was defined as "2 or 3 times a month" [20]. Cronbach α of the total scale was 0.84 [21].

Four questions were used to identify whether there is a culture of bullying at school. These questions are included in the Olweus BVQ. The questions were: “How often do teachers or other adults at school try to put a stop to it when a student is being bullied at school?, How often do other students try to put a stop to it when a student is being bullied at school?, Has any adult at home contacted the school to try to stop the student being bullied at school in the past couple of months?, When you see a student in your age being bullied at school, what do you feel or think?”

The self-reported Arabic version of the Strength and Difficulties Questionnaire (SDQ) was used to determine behavioral and emotional disorders. It is a brief behavioral screening questionnaire of approximately 25 attributes. The 25 attributes are divided into 5 subscales with 5 items in each one: emotional problems, conduct problems, hyperactivity-inattention, peer problems, and pro-social behavior. Each subscale ranges from 0 to 10. The total difficulties score is generated by summing the scores from all the subscales except the last one (pro-social behavior), creating a range from 0 to 40. Cronbach α for the total score is 0.87 [22]. Higher scores indicate more problems. The cut-off point of the SDQ was 20 for adolescents.

### Data analysis

Data analysis was performed using IBM SPSS Statistics Version 20.0. Data were summarized using means with standard deviation (SD) for quantitative variables and frequencies and percentages for qualitative ones. Comparison between groups was conducted using the chi-square or Fisher test for qualitative variables, and the T-student test for quantitative variables.

Finally, a multivariable analysis using logistic regression was performed to identify the factors associated with bullying victimization. The variables included in this model were those associated with bullying victimization with a p-value <0.20 in univariate analysis. We used a manual top-down step-by-step strategy: the variables finally retained in the model are those significant at the 5% threshold.

### Ethical considerations

This study was approved by a local Institutional Research Board under the reference number “IRB 00008931”. A written agreement from the local education directorate and permissions from all selected middle school directors were obtained. After obtaining written parental informed consent, verbal consent was obtained from the students accepting to participate in this study. Participation was voluntary. All the obtained data were treated anonymously and confidentially. Students were assured that their responses would not be disclosed to their teachers, parents, or anyone else, except the researchers.

## Results

### Prevalence of bullying victimization

A total of 1111 middle school students aged 11 to 17 years with a mean age of 13 years (SD = 1.03) were enrolled in this survey. The sample consisted of 472 males (42%) and 639 females (58%). Prevalence of bullying victimization was 45.8% (95%CI = 45.5–46.0). Verbal bullying was the most common type of victimization (29.5%), followed by physical victimization (22.5%), relational victimization (22.2%), and cyber victimization (14.3%). Bullying mostly occurred in the playground or athletic area (30.3%) and on the way to and from home (21.1%).

### Factors associated with bullying victimization: Univariate analysis

Table 1 shows the association between BV and the socio-demographic characteristics. BV was significantly higher among students living with other members of the family (grandparents, uncles etc…) compared to those living with both parents (respectively, 90.9% vs. 44.4%, p = 0.01). The mother’s education level was significantly inversely associated with VB (p = 0.02). When the mother’s educational level increased, the risk of being a bullying victim decreased. A significant association between BV and parents’ work status was also noted. The proportion of victims was higher among students with a working father (p = 0.01) or mother (p = 0.03). BV was significantly higher among repeating students than new ones (respectively, 57.8% vs. 43.3%, p = 0.00). Nevertheless, being a smoker seemed to protect against being a bully victim (p = 0.00).

**Table 1:** Socio-demographic and academic characteristics associated with bullying victimization among adolescents, El Kef, Tunisia.

Variables	Not victim N = 602 n (%)	Victim N = 509 n(%)	OR [95%CI]	*p*-value
**Gender (Male vs Female-reference)**				
Male	266(56.4)	206(43.6)	0.85[0.67–1.09]	0.21
Female	336(52.6)	303(47.4)	REF	
**Birth order**				
First	207(58.1)	149(41.9)	REF	
Middle	168(52.3)	153(47.7)	1.26[0.93–1.71]	0.12
Last	227(52.3)	207(47.7)	1.26[0.95–1.68]	0.10
**Place of residence**				
Urban	349(54.9)	287(45.1)	0.93[0.73–1.19]	0.59
Rural	253(53.3)	222(46.7)	REF	
**Whether living with both parents or not**				
Both parents	550(55.6)	440(44.4)	REF	
Single parent	51(46.4)	59(53.6)	1.44[0.97–2.14]	0.06
Another situation	1(9.1)	10(90.9)	12.5[1.59–98.02]	0.01
**Father’s education level**				
No education	43(47.8)	47(52.2)	0.90[0.79–1.02]	0.11
Primary	126(53.6)	109(46.4)		
Secondary	223(53.1)	197(46.9)		
University	210(57.4)	156(42.6)		
**Mother’s education level**				
No education	69(41.6)	97(58.4)	0.88[0.79–0.98]	0.02
Primary	161(55.7)	128(44.3)		
Secondary	162(59.3)	111(40.7)		
University	210(54.8)	173(45.2)		
**Father’s working status**				
Not working	16(94.1)	1(5.9)	REF	
working	586(53.6)	508(46.4)	13.87[1.83–104.95]	0.01
**Mother’s working status**				
Not working	392(56.6)	300(43.4)	REF	
working	210(50.1)	209(49.9)	1.3[1.02–1.65]	0.03
**School grade**				
7^th^	216(53.1)	191(46.9)	REF	
8^th^	243(53.9)	208(46.1)	0.96[0.74–1.26]	0.81
9^th^	143(56.5)	110(43.5)	0.87[0.63–1.19]	0.38
**Grade repetition**				
Yes	81(42.2)	111(57.8)	1.79[1.3–2.45]	0.00
No	521(56.7)	398(43.3)	REF	
**Smoking**				
Yes	25(37.3)	42(62.7)	0.48[0.28–0.80]	0.00
No	577(55.3)	467(44.7)	REF	
**Alcohol consumption**				
Yes	3(33.3)	6(66.7)	0.42[0.10–1.68]	0.22
No	599(54.4)	503(45.6)	REF	
**Substance use**				
Yes	4(30.8)	9(69.2)	0.37[0.11–1.21]	0.10
No	598(54.5)	500(45.5)	REF	
**Practice of leisure activity**				
Yes	449(55.9)	354(44.1)	REF	
No	153(49.7)	155(50.3)	1.28[0.98–1.67]	0.06

### Factors associated with bullying victimization: Multivariate analysis

A logistic regression model was applied to explore the factors associated with BV (Table 2). Significant factors detected by the uni-variable analysis have been introduced into the model (e.g., grade repetition, birth order, whether living with both parents or not, father’s education level, mother’s education level, father’s working status, mother’s working status, smoking, alcohol consumption, substance use, practice of pleasure activity). The last step of the model revealed that repeating a grade (OR = 1.82, 95%CI = 1.31–2.54), having a working father (OR = 17.68 95%CI = 2.29–136.15), and having a working mother (OR = 1.88, 95%CI = 1.39–2.53) were the factors significantly associated with bullying victimization. Nevertheless, a higher mother’s educational level (OR = 0.76, 95%CI = 0.67–0.88) was a protective factor against bullying victimization.

**Table 2:** Predictors of bullying victimization among adolescents, El Kef, Tunisia: Binary logistic regression model.

Predictors	OR [95%CI]	*p-*value
**Grade repetition** Yes No*	1.82 [1.31–2.54]	<10⁻^3^
**Mother’s education** No education Primary Secondary University	0.76 [0.67–0.88]	<10⁻^3^
**Father’s working status** Working Not working*	17.68[2.29–136.15]	<10⁻^3^
**Mother’s working status** Working Not working*	1.88[1.39–2.53]	<10⁻^3^

* Reference variable in multivariable analysis.

### Bullying culture

A key component of bullying culture is the students’ perceptions regarding whether other students or their teachers try to prevent bullying behavior or not. While few students reported that teachers frequently tried to stop bullying at school (almost always”: 14%; “often”: 9.4%), over 55% of the students reported that teachers only “once in a while” (20.3%) or “almost never” (35.5%) tried to stop bullying (Table 3).

**Table 3:** Bullying culture among teachers, students, and family in El Kef, Tunisia.

Questions	n	%
**How often do teachers or other adults at school try to put a stop to it when a student is being bullied within the school?**		
almost never	394	35.5
Once in a while	225	20.3
Sometimes	223	20.1
Often	104	9.4
Almost always	155	14.0
**How often do other students try to put a stop to it when a student is being bullied within the school?**		
almost never	489	44.0
Once in a while	193	17.4
Sometimes	225	20.3
Often	83	7.5
almost always	111	10.0
**Has any adult at home contacted the school to try to stop a student being bullied within the school in the past couple of months?**		
No, they haven’t contacted the school.	425	85.5
Yes, they have contacted the school once.	51	10.3
Yes, they have contacted the school several times.	21	4.2
**When you see a student in your age being bullied within the school, what do you feel or think?**		
That is probably what he or she deserves.	97	8.7
I don’t feel anything.	109	9.8
I feel a bit sorry for him or her.	192	17.3
I feel sorry for him or her and want to help him or her.	713	64.2

Most of the students (61.4%) reported that other students rarely tried to stop bullying at school (“once in a while”: 17.4%; “almost never”: 44%). Only 72 students (14.5%) stated that at least one family member contacted the school once or several times in an attempt to stop bullying. One more aspect of bullying culture is whether students lack empathy for victimized students or not. Hence, the results showed that 64.2% of the included students endorsed the empathic response “I feel sorry for him or her and go to help him or her”.

### Bullying and emotional and behavioral disorders

Behavioral and emotional disorders were assessed using the first four domains of the SDQ. The cut-off point of the disorders was 20. The findings showed that 517 students (46.5% 95%CI = 43.6–49.4) had mental health difficulties. The association between BV and emotional as well as behavioral problems among students is presented in Table 4. Significant differences between victims and non-victims were noted in all individual subscales and the total score of difficulties (all p <0.01).

**Table 4:** Bullying victimization associated with emotional and behavioral disorders.

Variables	Not victim (m±SD)	Victim (m±SD)	*p*-value
**Emotional problems**	3.62±2.44	5.02±2.46	<0.01
**Conduct problems**	3.33±1.70	4.09±1.77	<0.01
**Hyperactivity/inattention**	5.25±1.71	5.65±1.77	<0.01
**Peer problems**	5.26±1.75	6.10±1.62	<0.01
Total difficulties score	17.46±5.30	20.86±5.06	<0.01
**Pro-social behavior**	7.77±2.16	7.60±2.22	0.20

m: Mean; SD: Standard deviation.

## Discussion

This study investigated the prevalence and the factors associated with BV within schools in Tunisia and its association with mental health outcomes (emotional and behavioral disorders). The current findings showed that the prevalence of bullying behavior among middle school adolescents was high (45.8%). This finding is inconsistent with a previous research reporting a prevalence of 11.3% of bullying victims in Sousse, Tunisia [9] but similar to the mean prevalence reported for north african countries (42.7%) [6]. According to the literature, BV rates vary across countries. It ranges between 5% and more than 70% [9,11,23,24].

High rates of BV among adolescents are reported in some Arab countries, such as Egypt (57%) and Lebanon (46.5%) [11,22]. The prevalence of BV in high-income countries, such as Canada (25.2%) is lower than in lower-middle-income countries (e.g., Egypt, Lebanon, Tunisia) and it is higher compared to upper-middle-income countries, such as Malaysia (16.2%) [23,24]. This variation in prevalence between countries could be attributed to methodological and cultural differences in defining the problem and in the assessment instruments [25]. It could also be attributed to the differences in national policies and preventive intervention programs applied to reduce this social evil.

The current study showed that verbal bullying was the most common type of bullying. This is consistent with the findings from a Jordanian study [26] revealing that verbal bullying is the most common among adolescents. The high prevalence of this form of bullying could be explained by the difficulty to be identified by schoolteachers. In addition, students are often aware of the rules prohibiting physical harm to others [27].

Playgrounds and the way to school were the most common places where bullying occurred. Similarly, previous research reported that sites within the school environment (e.g., cafeteria, playground, and hallways) are the most common settings of bullying [28]. This suggests that bullying behavior occurs in places without adult supervision, such as playgrounds, hallways, and the way to and from school.

Regarding the socio-demographic and academic factors associated with BV, the prevalence of being a victim was significantly higher among students failing in the previous school year. Similarly, a previous study suggested that old-for-grade status is significantly related to more victim behavior compared to age-appropriate-for-grade [29]. This could be explained by the fact that students repeating a class had problems in getting involved in a new class. They were more likely to be rejected and neglected by their new classmates [30]. In the current study, the finding that maternal education was inversely associated with BV is consistent with a previous research conducted in rural Egypt among preparatory and secondary school students [11]. Similarly, Jansen et al. found that higher parental education is a protective factor against bullying and that the parents’ low educational level is independently associated with bullying [31,32]. This can be attributed to the fact that level of education can have an impact on socio-economic status, as well as on children’s behavior and lifestyle.

Regarding parental work status, students with a working father or mother were respectively 17.68 times or 1.88 times at a higher risk of being bullied. This finding is partially consistent with the literature. Margklara et al. found that among Greek students, bullying victims are significantly more likely to have unemployed mothers [33]. Another study found that long hours of mothers’ work increase the likelihood of bullying behaviors; however, fathers’ work hours do not predict bullying behaviors [23]. These findings were explained by the fact that working mothers are too busy to talk to their children on daily basis. Non-working mothers could be more available and responsive to talk to their children and help them deal with problems in daily life. The explanation regarding the father’s work goes with the Tunisian context. Thus, the status of working parents can be harmful to children. Parents must therefore be conscious of this fact by devoting more time to listen to their children.

Concerning peer response to BV, most of the students reported that their classmates rarely try to stop bullying. This finding is consistent with a Lebanese study conducted by Khamis among school-age children in the great Beirut area [22]. This finding could be explained by the fact that the culture of bulling shapes bystanders’ behavior by encouraging passivity [22,29].

Very few parents were aware and they react to their children’s victimization. This reflects that bulling could happen without a persuasive intervention from the victim’s parents. Previous research showed that intervention and stable support from parents, including listening and offering affection, trust, and respect is particularly important for bullied adolescents.

Only around two-thirds of students displayed empathy for the victims. This result is similar to that found in a previous research conducted among Lebanese students [22], reporting he presence of a common set of normative beliefs that promote bullying. Therefore, interventions against bullying should target peer bystanders to enhance their empathy toward victims [34].

BV does not only affect the students’ physical status but also induces mental health problems. The SDQ tool used in this study revealed that all difficulty dimensions’ scores (hyperactivity, emotional scale, conduct problems scale, and peer problems scale) were significantly high among victims. Similarly, an Egyptian study reported that bully victims have significantly higher scores on the total score of SDQ and in the emotional, conduct, and hyperactivity problems [11].

Over the past two decades, researchers have conducted a number of systematic reviews to investigate the relationship between bullying victimization and mental health disorders. The most recent ones concluded that there is strong evidence for causal associations between bullying victimization and mental health problems, such as depression, anxiety, general ill health, and suicidal ideation and behavior. In addition, there is a possible causal link between bullying and tobacco as well as illicit drug use [3].

This study aimed to determine the prevalence and the factors associated with BV among a large random sample of middle school students in Tunisia. The large sample involved in this study increases the precision of our estimates and the power of the present study to draw solid conclusions. To the best of the authors’ knowledge, it is the first Tunisian study exploring the association of BV with mental health outcomes. The current findings support the growing literature emphasizing the harmful effects of BV in school-age adolescents, regardless of cultural background.

Nevertheless, this study has some limitations that should be considered. Indeed, the current findings are based on a cross-sectional design, which makes it impossible to draw causal interpretations. Moreover, self-reported scales were used, which can lead to reporting bias.

### Recommendations

Students have the right to live in an educational environment that is safe and free from bullying. Many countries have developed numerous strategies to tackle bullying, including national policies and legislation, as well as more prevention programs.

Among the various school anti-bullying programs or whole-school interventions described in the literature, the Olweus Bullying Prevention Program (OBPP) stands out as a universal initiative that has consistently been more successful in reducing the number of bullies and is among the best methods of reducing victimization, particularly in primary, middle and high schools [35]. The OBPP aims to restructure the school environment and addresses the problem of bullying at three levels: school-wide, classroom and individual. These interventions combined classroom rules, lectures addressing bullying, activities with bullies/victims/bystanders, information provided to parents, increased supervision, disciplinary methods, cooperation between researchers and the school staff, training of teachers, and technological resources [36].

The design and implementation of anti-bullying school programs in Tunisia to reduce the harmful effects of bullying is becoming a necessity given the increasing prevalence of bullying.

Since these whole school interventions cannot be easily established, selective interventions targeting bullied individuals such as social skills training (SST) can be considered in the meantime [37].

## Conclusions

Bullying victimization is a serious and major public health issue that has a negative impact. It is negatively associated with adolescents’ well-being and it therefore requires special attention in schools. This study showed that the prevalence of bullying victimization in middle schools was high. It was associated with grade repetition, mother’s educational level, mother’s and father’s employment, and smoking. BV significantly led to behavioral and emotional disorders, including emotional problems, conduct problems, peer problems, and hyperactivity/inattention. National policies for bullying prevention within schools are potentially needed. Improving students’ problem-solving and soft skills is also essential.

## Supporting information

S1 File (XLSX)
